# Double-sieving-defective aminoacyl-tRNA synthetase causes protein mistranslation and affects cellular physiology and development

**DOI:** 10.1038/ncomms6650

**Published:** 2014-11-27

**Authors:** Jiongming Lu, Martin Bergert, Anita Walther, Beat Suter

**Affiliations:** 1 Institute of Cell Biology, University of Bern, Baltzerstrasse 4, Bern 3012, Switzerland

## Abstract

Aminoacyl-tRNA synthetases (aaRSs) constitute a family of ubiquitously expressed essential enzymes that ligate amino acids to their cognate tRNAs for protein synthesis. Recently, aaRS mutations have been linked to various human diseases; however, how these mutations lead to diseases has remained unclear. In order to address the importance of aminoacylation fidelity in multicellular organisms, we generated an amino-acid double-sieving model in *Drosophila melanogaster* using phenylalanyl-tRNA synthetase (PheRS). Double-sieving-defective mutations dramatically misacylate non-cognate Tyr, induce protein mistranslation and cause endoplasmic reticulum stress in flies. Mutant adults exhibit many defects, including loss of neuronal cells, impaired locomotive performance, shortened lifespan and smaller organ size. At the cellular level, the mutations reduce cell proliferation and promote cell death. Our results also reveal the particular importance of the first amino-acid recognition sieve. Overall, these findings provide new mechanistic insights into how malfunctioning of aaRSs can cause diseases.

## Abstract (web summary)

Accurate loading of amino acids to their cognate tRNA is essential to avoid mistranslation during protein synthesis, which has been linked to human diseases. Here, Lu *et al*. present a *Drosophila* model that demonstrates the necessity of two distinct ‘sieves’ to ensure accurate amino acid loading for proper development.

## Graphical abstract

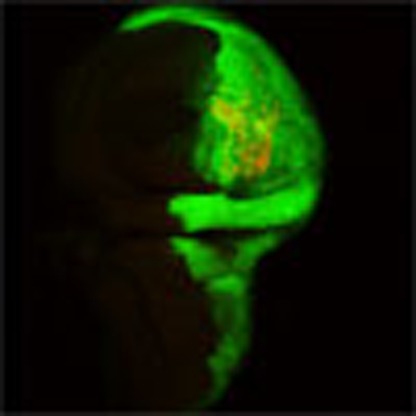

The faithful transfer of biological information from DNA to protein is critical for the development, physiology, survival and reproduction of all organisms. A key step in ensuring the proper decoding of the genetic information is performed by the aminoacyl-tRNA synthetases (aaRSs)^1^. During aminoacylation, aaRSs link the cognate amino acid to the transfer RNA (tRNA). This ligation reaction is a two-step process, where the amino acid is first activated by ATP to form an aminoacyl adenylate (aa-AMP) intermediate, followed by the transfer of the activated aa-AMP to the adenosine residue located at the 3′ end of the tRNA—forming the aa-tRNA^2^,^3^. aa-tRNAs are then delivered to the ribosome where the transfer of the amino acid to the nascent polypeptide chain occurs. In this set of events the proper charging of the tRNA with its cognate amino acid is crucial for the flawless translation of the genetic code into the protein sequence.

Recent studies have linked aaRSs with different human diseases^4^,^5^. Mutations in cytoplasmic glycyl-tRNA synthetase (GlyRS), tyrosyl-tRNA synthetase (TyrRS), lysyl-tRNA synthetase (LysRS) and alanyl-tRNA synthetase (AlaRS) have been shown to be associated with Charcot-Marie-Tooth (CMT) disease, one of the most common inherited neurological disorders^6^,^7^,^8^,^9^. However, how these mutations relate to the disease phenotypes has remained unclear, although impaired aminoacylation activity and loss of non-canonical functions of the aaRSs have been proposed as possible mechanisms^4^,^5^,^10^. Here we describe a fine-tuning mechanism of aminoacylation accuracy, the failure of which can dramatically reduce translation fidelity and affect cellular function.

The accuracy of aminoacylation first depends on the correct recognition of the appropriate amino acid by an aaRS^11^,^12^,^13^. Structural differences between amino acids prevent non-cognate amino acids from binding to the aminoacylation activation site. However, the task of distinguishing between two very similar amino acids becomes challenging for aaRSs and can lead to errors. If non-cognate amino acids are activated, aaRSs employ an editing mechanism to hydrolyse the misactivated aa-AMP (pre-transfer editing) or misacylated aa-tRNA (post-transfer editing)^14^,^15^,^16^. The accuracy of charging the tRNA is therefore ensured at two levels: amino-acid selection and editing of errors, leading to the term ‘double-sieve’ model^17^,^18^. The first sieve—amino-acid recognition—is a ‘coarse sieve’ that excludes most non-cognate amino acids; the second sieve—amino-acid editing—is a ‘fine sieve’ that hydrolyses the structurally similar, non-cognate amino acids. The first sieve is an essential feature of every aaRS, while the second sieve has so far been found in about half of the aaRSs^19^. In recent years, significant progress has been made in elucidating the structural basis and molecular mechanisms of the double sieve in single cell organisms^19^; however, double-sieving animal models are still lacking.

Phenylalanyl-tRNA synthetase (PheRS) is an (αβ)_2_ heterotetramer^20^,^21^ that possesses both sieving mechanisms. Phenylalanine (Phe) is the cognate amino acid for PheRS; however, tyrosine (Tyr, 4-hydroxyphenylalanine) can also be charged at low frequency^22^. The C terminus of the α-subunit forms the core structure for the aminoacylation function together with domain B6-7 of the β-subunit and this structure serves as the amino-acid recognition sieve^12^,^23^. The second sieve is capable of post-transfer editing and is built by domain B3-4 of the β-subunit^24^. While studies in bacteria and archaea have characterized the molecular mechanism of the sieves^24^,^25^,^26^, the physiological and pathological roles still needed to be addressed in a multicellular eukaryotic model.

Using *Drosophila*, we present the first double-sieving animal model for PheRS. We studied the individual and combined effects of mutations in the amino-acid recognition and -editing sites of PheRS to elucidate the importance of these two sieves in different tissues and stages of the fly life cycle. Sieving defects lead to neurodegeneration-related phenotypes, advanced ageing and reduced organ size. We further assessed whether reduced cell proliferation and enhanced apoptosis can cause the observed defects and whether, at the molecular level, the sieving defects cause protein mistranslation and endoplasmic reticulum (ER) stress. This double-sieving fly model provides novel insights into the mechanisms underlying aaRSs-related diseases.

## Results

### *PheRS* mutants and mutual stabilization of subunits

**Figure 1:**
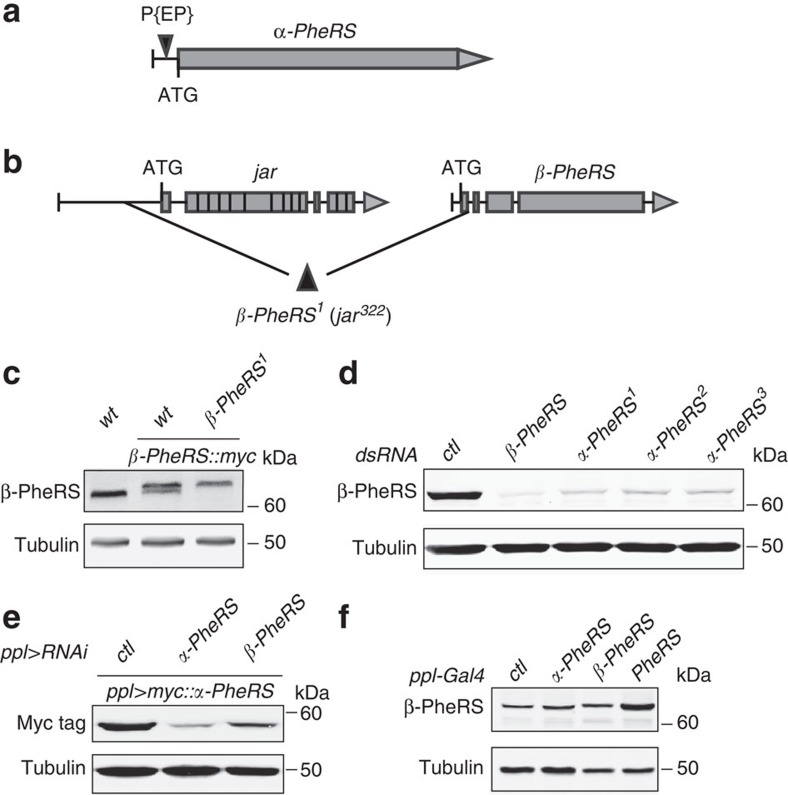
*Drosophila PheRS* loci and mutual stabilization of the two subunits. (**a**) The P{EP}-element insertion allele *G2060* is an *α-PheRS* mutant. (**b**) *β-PheRS*^*1*^ deletes the first exon of *β-PheRS* and the neighbouring *jar* gene. The scheme is on the basis of the information available on FlyBase and is not to scale. (**c**) Expression of β-PheRS variants in *β-PheRS*^*1*^ and wild-type fly heads. The lower band is wild-type β-PheRS, and the upper band is the myc-tagged β-PheRS. (**d**) β-PheRS protein levels in Kc cells upon knockdown of the subunits. The control (*ctl*) is *Amp*^*R*^ RNAi. *α-PheRS*^*1*^, *α-PheRS*^*2*^ and *α-PheRS*^*3*^ stand for three different dsRNAs against *α-PheRS.* Knockdown of either subunit downregulates β-PheRS levels. (**e**) Myc::α-PheRS protein levels in fly fat bodies upon knockdown of the subunits. *ppl-Gal4* drove *myc::α-PheRS* expression and RNAi against either subunit. The control (*ctl*) is *Cdk7* RNAi. Knockdown of either subunit downregulates Myc::α-PheRS levels. (**f**) Protein levels upon *PheRS* overexpression in the fat body dissected from wandering third instar larvae. *ppl-Gal4* was used as driver and the control (*ctl*) is GFP overexpression. Higher levels of protein accumulated only when both subunits (*PheRS*) were co-overexpressed.

*Drosophila* cytoplasmic PheRS is encoded by the X chromosomal *α-PheRS* and the third chromosomal *β-PheRS*. A P-element insertion in the 5′-untranslated repeat of the *α-PheRS* transcript (Fig. 1a) causes recessive lethality that can be rescued by a genomic copy of *α-PheRS* (*gαPheRS*). *jar*^*322*^ deletes the promoter region and the first exon of *β-PheRS* as well as the neighbouring *jar* gene^27^ (Fig. 1b). We named it *β-PheRS*^*1*^ because a genomic copy of *β-PheRS* (*gβPheRS*) can rescue its lethality. Rescuing the lethality of hemizygous *PheRS*^*1*^ animals with an *myc*-tagged genomic copy of *β-PheRS* (*gβPheRS::myc*) allowed us to assess levels of PheRS produced from the *β-PheRS*^*1*^ allele. As shown in Fig. 1c, β-PheRS antibodies only detected a single band corresponding to the larger β-PheRS::myc, indicating that *β-PheRS*^*1*^ is indeed a null allele. Furthermore, the rescue of the null mutant shows that the tagged protein is at least partially functional.

*β-PheRS* RNA interference (RNAi) knockdown in *Drosophila* Kc cells dramatically decreased its protein level (Fig. 1d). Interestingly, three different *α-PheRS* RNAi treatments also downregulated β-PheRS protein levels, indicating that the α-subunit stabilizes the β-subunit. To study the reciprocal relationship in the absence of an α-PheRS antibody, we expressed *myc::α-PheRS* and the RNAi constructs simultaneously in the larval fat body. In this assay, RNAi knockdown of the α- or β-PheRS subunit decreased Myc::α-PheRS protein levels (Fig. 1e), indicating that the β-subunit also stabilizes the α-subunit. Therefore, the two subunits normally stabilize each other. This interpretation is further supported by a tissue-specific overexpression experiment. Single overexpression of either α-PheRS or β-PheRS had only minor effects on the accumulation of β-PheRS, while co-overexpression of both subunits strongly increased β-PheRS protein levels (Fig. 1f). The mutual stabilization of the two subunits allowed us to investigate the dominant-negative effect of mutations for one subunit by using the *UAS-Gal4* system. If the total amount of enzyme stays at normal levels, the overproduction of the mutant subunit makes this the predominant variant in the cell.

### PheRS amino acids crucial for aminoacylation fidelity

**Figure 2:**
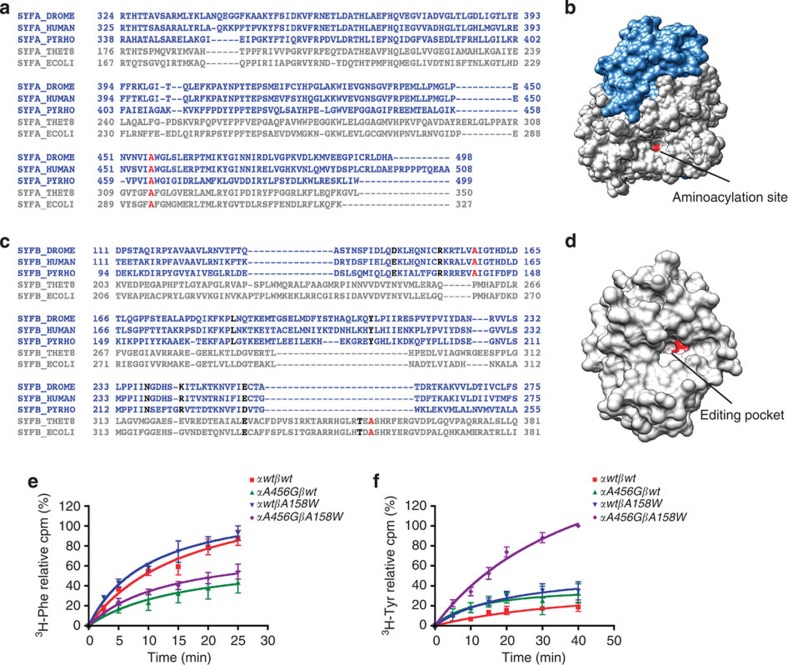
Sieving-defective mutations in *Drosophila* PheRS. (**a**,**c**) Structure-based alignment of PheRS sequences using the PROMALS3D method. The sequences of prokaryotes and archaea/eukaryotes are shown in grey and blue, respectively. The C-terminal region of α-PheRS is listed in **a**, and the red Ala is the amino-acid residue substituted by Gly. Domain B3-4 of β-PheRS is shown in **c**. Residues crucial for editing, the ones at the entrance of the editing pocket (red) and the ones at the activation centre (black), are indicated. DROME, *Drosophila melanogaster*; HUMAN, *Homo sapiens*; PYRHO, *P. horikoshii*; THET8, *T. thermophilus*; ECOLI, *E. coli*. (**b**,**d**) Structure of the *Drosophila* PheRS catalytic module (first sieve) and domain B3-4 (second sieve) modelled by UCSF Chimera. The residues highlighted in red in **b**,**d** correspond to the red Ala in **a**,**c**, respectively. The catalytic module (**b**; domain B6-7 in light blue and domain A1-2 in grey) was modelled on the human PheRS (PDB: 3L4G). Domain B3-4 was modelled on *P. horikoshii* PheRS (**d**; PDB: 2CX1). (**e**,**f**) The Phe and Tyr aminoacylation activities of PheRS variants were determined *in vitro*. (**e**) PheRS variants are still active in Phe aminoacylation. However, while the enzymatic activity of the *βA158W* mutant is normal, the activity of the α*A456G* mutant is somewhat reduced. (**f**) Tyr mis-aminoacylation activity of PheRS variants. Wild-type PheRS produced very low levels of TCA-insoluble Tyr and the single mutant αA456G and βA158W produced only slightly elevated levels. In contrast, the αA456G, βA158W ‘double-mutant’ protein dramatically misacylated Tyr. Values are the means of three independent experiments. Error bars show s.d.

On the basis of previous results from *Escherichia coli*, *Thermus thermophilus* and *Pyrococcus horikoshii*^24^,^25^,^26^, we first predicted potential crucial amino-acid residues for aminoacylation fidelity in *Drosophila* PheRS. We used the knowledge about the secondary structure to align the sequences for both subunits of the selected prokaryotic and archaeal/eukaryal PheRS^28^. The sequences are highly conserved within the archaea/eukaryotes and more diverse between the bacterial and archaeal/eukaryal species (Fig. 2a–d). Proper Phe specificity for the activation step is controlled by the amino-acid recognition sieve. A294 in *E. coli* and A463 in *P. horikoshii* are the key residues in α-PheRS to ensure substrate specificity because they restrict the space of the pocket to the size of Phe^12^. Replacing this Ala by the smaller Gly enlarges the Phe-binding pocket, making it more permissive to receive the non-cognate Tyr, thereby enhancing Tyr misactivation. Despite the high sequence divergence between the bacterial and the archaeal/eukaryal α-subunits, we identified the highly conserved A456 in *Drosophila* α-PheRS as the homologous position (Fig. 2a,b). To produce a PheRS enzyme with an increased activation error rate, we therefore introduced the *A456G* mutation into the *Drosophila α-PheRS* gene.

The safeguard mechanism for accurate aminoacylation is the editing pocket located in domain B3-4 of the β-subunit. For proper editing, amino acids in two regions are considered to be crucial. The first are the amino acids at the entrance of the editing pocket, and the second are the amino acids at the activation centre. According to the well-studied archaea *P. horikoshii*^25^, we identified the corresponding amino-acid residues in *Drosophila* (Fig. 2c,d). Ala141 in *P. horikoshii*, corresponding to Ala158 in *Drosophila*, sits at the entrance site of the editing pocket and is considered to be the key residue for the editing function. Mutating A158 to the biggest amino acid, Tryptophan, may plug the editing pocket, thereby preventing Tyr from entering and being edited. We therefore introduced this mutation in *Drosophila β-PheRS*. These two mutations should reveal the importance of the two sieving functions in a complex animal model.

Introduced into the fly genome, the genomic wild-type *β-PheRS* and the *β-PheRS*^*A158W*^ mutant transgenes rescued the *β-PheRS*^*null*^ lethality well. Surprisingly, the *β-PheRS*^*null*^ flies rescued with the genomic *β-PheRS*^*A158W*^ mutant construct (*gβA158W*) did not reveal any obvious phenotypes, but, instead, showed normal developmental time, body size and bristle shape, indicating that under standard laboratory conditions this mutation is not detrimental. The results with the *α-PheRS* mutations are less straightforward to interpret. Already the wild-type genomic rescue construct of *α-PheRS* showed only a weak rescue activity in the background of the lethal *α-PheRS* mutant, suggesting that the genomic rescue construct may be lacking some important sequences. It was therefore not surprising that the mutant genomic *α-PheRS*^*A456G*^ (*gαA456G*) could not rescue this lethality. In order to test the function of α-PheRS and the combined effect of the two mutants, we therefore resorted to the dominant-negative approach of overexpressing the α-PheRS variants using *UAS-Gal4* (ref. 29).

We also examined whether the *Drosophila* αA456G and βA158W mutant proteins are still capable of performing aminoacylation and, if so, whether the mutations affect the sieving functions and increase the error rate. Wild-type and different mutant proteins were expressed in *E. coli* and purified. The wild type and the βA158W mutant showed the highest activities in Phe aminoacylation (Fig. 2e). The αA456G mutant protein showed somewhat decreased enzymatic activity regardless of whether it was in combination with wild-type or mutant β-PheRS. Despite the observed reduction mutant α-PheRS was clearly still active. We also tested the ability of these proteins to mischarge tRNA^Phe^ with the non-cognate Tyr (Fig. 2f). Wild-type PheRS converts only very little Tyr into trichloroacetic acid (TCA)-insoluble material, while the single mutants αA456G or βA158W exhibited similar but slightly higher activities. In contrast, the αA456G, βA158W double-mutant enzyme showed a dramatically increased misacylation with Tyr, demonstrating that this mutant fly PheRS is indeed sieving defective. We therefore named these alleles *PheRS sieving-defective* (*PheRS-sd*) and started investigating their phenotypes in different tissues and stages.

### *PheRS-sd* mutations in the eye result in retinal defects

**Figure 3:**
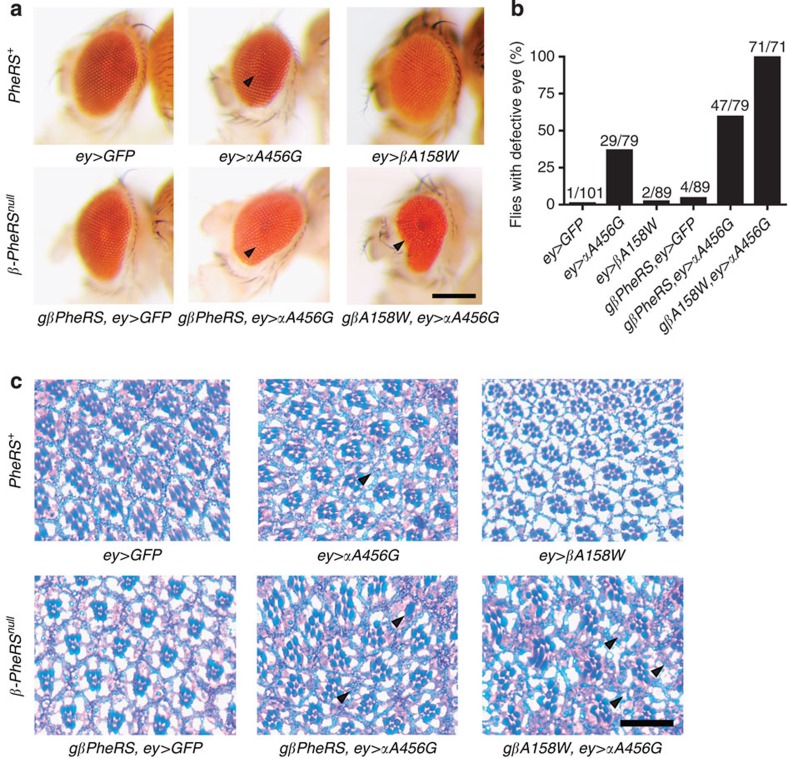
Expressed in the eye *PheRS-sd* mutations cause retinal defects. (**a**) Eye surface phenotypes induced by *ey-Gal4* (*ey*)**-driven expression of *PheRS-sd* mutations. In the *PheRS* wild-type background, GFP expression served as control. Some ommatidia in αA456G-expressing eyes (arrowhead) are not properly organized, while βA158W-expressing ones are normal. Flies expressing GFP or αA456G in the *gβPheRS*-rescued *β-PheRS*^*null*^ background were similar to the ones in the *PheRS*^*+*^ wild-type background. Flies expressing αA456G in the *gβA158W* background gave rise to severe rough eye phenotypes. Flies were ~1-week old and the scale bar represents 200 μm. (**b**) Quantification of eye defects from the experiment shown in **a**. If ommatidia showed irregularities in any region of either eye, the animal was recorded as defective. Flies were ~1-week old. (**c**) Light microscopic images of semithin retina sections. Hexagonal units are individual ommatidia in which seven photoreceptor cells are visible in a given plane. Well-oriented ommatidia with normal complements of photoreceptors are seen in controls and in βA158W-expressing eyes. The expression of αA456G alone or αA456G together with βA158W resulted in reduced numbers of photoreceptor cells in individual ommatidia (indicated by arrowheads) and a misaligned pattern. Scale bar represents 10 μm.

The *Drosophila* compound eye is composed of several hundred identical ommatidia units that form a highly regular surface. This makes it a very sensitive structure to identify irregularities in cell proliferation, cell differentiation and cell death^30^. In addition, it is a good organ to study neurodegeneration because the photoreceptor cells are neuronal cells that are not essential for fly viability^31^. We therefore assessed the effect of *PheRS-sd* mutations on the eyes by driving the transgenes with *eyeless-Gal4* (*ey-Gal4*) (Fig. 3a). Eyes expressing the *αA456G* mutant *α-PheRS* mostly displayed a regular surface as the normal eye, although a few ommatidia were not properly organized in certain regions. When expressing the β-subunit mutation *βA158W*, we did, however, not observe any defects, indicating that this mutation does not cause major defects on its own. Next, we tested for combined effects of the two mutations. In the *β-PheRS*^*null*^ background rescued with the *gβA158W* mutant transgene, the expression of the *αA456G* mutant caused a very severe disruption of the eye organization. Most ommatidia became irregular, the entire eye was smaller and the eye surface became rough. In contrast, when the *β-PheRS*^*null*^ mutant was rescued with *gβPheRS*, the expression of the mutant *αA456G* showed a similar weak phenotype as when this mutant was expressed in the wild-type background. We also quantified the frequency of animals with defective eyes for each genotype (Fig. 3b). Around half (40–60%) of the flies expressing the *αA456G* mutation showed ommatidia defects, and this frequency went up to 100% in flies expressing the *αA456G* plus *βA158W* mutations in the eye. In contrast, we only observed few defective eyes in controls and in the group expressing the *βA158W* mutation. These results together indicate that the proper double-sieving function of PheRS is required for eye development in *Drosophila*.

We also examined the eye defects in semithin sections (Fig. 3c). The retina of controls and *βA158W* mutants revealed regularly aligned ommatidia with regular shapes and normal number of photoreceptor cells. *αA456G* mutants looked different in both *β-PheRS*^*+*^ backgrounds. While most of the ommatidia were still normal, some individual ones lacked photoreceptors, causing these ommatidia to be smaller and the retina to display a misaligned pattern in some regions. A much stronger phenotype was found in eyes expressing *αA456G* plus *βA158W PheRS*. Their retina and ommatidia lacked regular patterns, shapes and organization because of smaller ommatidia and absence of many cells. Taken together, our results demonstrated that *PheRS-sd* mutations cause defects in the retina by affecting the number and shape of photoreceptors (and possibly other cell types). The fact that these mutations affect photoreceptors indicates that the double sieve is important in the neural tissue.

### Sieving-defective mutations impair locomotion and lifespan

**Figure 4:**
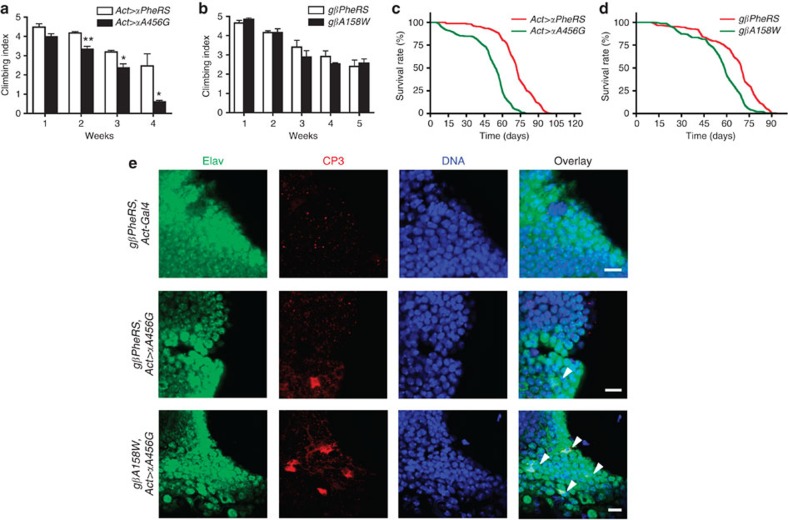
Sieving-defective mutations induce impaired locomotive performance and advanced ageing. (**a**,**b**) Negative geotaxis climbing assays were performed to study locomotive behaviour. For each genotype, three groups of male flies were tested at the indicated age. The assay was recorded as video, and the movies were analysed to determine the climbing index. (**a**) *αA456G* mutant flies displayed progressively decreasing climbing ability. Expression of the α-subunit (wild-type and *αA456G* mutant) was driven by *Act-Gal4*. (**b**) *βA158W* did not differ from the wild type during the first 5 weeks. Transgenes were in the *β-PheRS*^*null*^ background, rescued by *gβPheRS* or *gβA158W* under their endogenous promoter. Error bars show s.e.m. *n*=3, *P<0.05, **P<0.01, *t*-test. (**c**,**d**) Lifespan analysis of the male flies with the same genotype as in **a**,**b**. (**c**) *αA456G* mutant flies aged faster than wild type, and the differences became apparent already at early stages. *P*<0.001, log-rank test. (**d**) At younger age, *gβA158W* mutant flies aged similarly as the wild type, but at older age (more than 50 days old) the mutants aged faster. *P*<0.001, log-rank test. (**e**) Confocal microscopy images of the ventrolateral protocerebrum region of the adult fly brain. Embryos and larvae were kept at 18 °C to keep Gal80^ts^ active to repress Gal4. With this protocol, normal adults eclosed (while the ones expressing *αA456G* and *βA158W* were lethal without Gal80^ts^) and these flies were then incubated at 29 °C for ~7 days, inactivating Gla80^ts^ and activating Gal4. Elav (green) marks neuronal cells, and anti-CP3 stains apoptotic cells (red). A few CP3-positive cells were detected in neurons expressing the *αA456G* mutant, and many more apoptotic neurons (arrowheads) were observed when both *αA456G* and *βA158W* variants were expressed. DNA is in blue. Scale bars represent 5 μm.

To further study the long-term effect of *PheRS-sd* mutations, we analysed their locomotive performance by a negative geotaxis climbing assay. Ubiquitous expression of the wild-type or mutant α-subunit was driven by *Act-Gal4*, and the climbing capability was recorded once a week (Fig. 4a, Supplementary Movie 1). While *αA456G* flies showed a similar climbing ability as wild-type animals during the first week, they displayed clearly impaired locomotive performance during the subsequent weeks, and during the 4th week their climbing index decreased to about one quarter of the index of same age control flies. In contrast, the climbing ability of *βA158W* mutants did not differ significantly from wild-type controls (Fig. 4b). The *Act-Gal4*-driven expression of the *αA456G* in the *gβA158W* transgenic background turned out to be lethal during development, preventing us from examining the locomotive performance of adults.

Sieving-defective mutations might result in chronic cellular stress that affects the animal’s physiology. We therefore tested whether these mutations result in a shortening of the lifespan. For this purpose animals were grouped into 10 flies per vial and the dead events were recorded three times a week. Again, the *αA456G* mutants displayed a phenotype (Fig. 4c). Their mean lifespan was ~55 days, while the mean lifespan of the controls was over 70 days. In contrast, there were no clear differences in survival rates between younger flies (less than 50 days old) expressing either wild-type *β-PheRS* or the mutant *gβA158W*. However, the situation changed when flies became older. After the age of 50 days the *gβA158W* mutants aged faster than the wild-type controls (Fig. 4d). In summary, a defect in the first sieve of PheRS leads to progressive loss of locomotive ability and faster ageing, while a defect in the second sieve enhances ageing in older animals.

The enhanced locomotion problems with age and the reduced lifespan phenotype could at least partially be caused by neurodegeneration. To address the possibility that *PheRS-sd* mutations cause not only developmental defects but also adult neurodegeneration, we repressed the expression of the *αA456G* mutant until the adults eclosed using Gal80^ts^ (ref. 32). Several days after inactivating Gal80^ts^, adult brains were stained for Cleaved Caspase 3 (CP3) to identify apoptotic cells^33^. Expressing the *αA456G* mutant caused some Elav-positive brain cells (that is, neuronal cells^34^) to go into apoptosis; moreover, many more neuronal cells were CP3-positive when both, *αA456G* and *βA158W*, were expressed (Fig. 4e). These results show that *PheRS-sd* mutations induce neurodegeneration in adult animals.

### Organ growth is impaired in sieving-defective mutants

**Figure 5:**
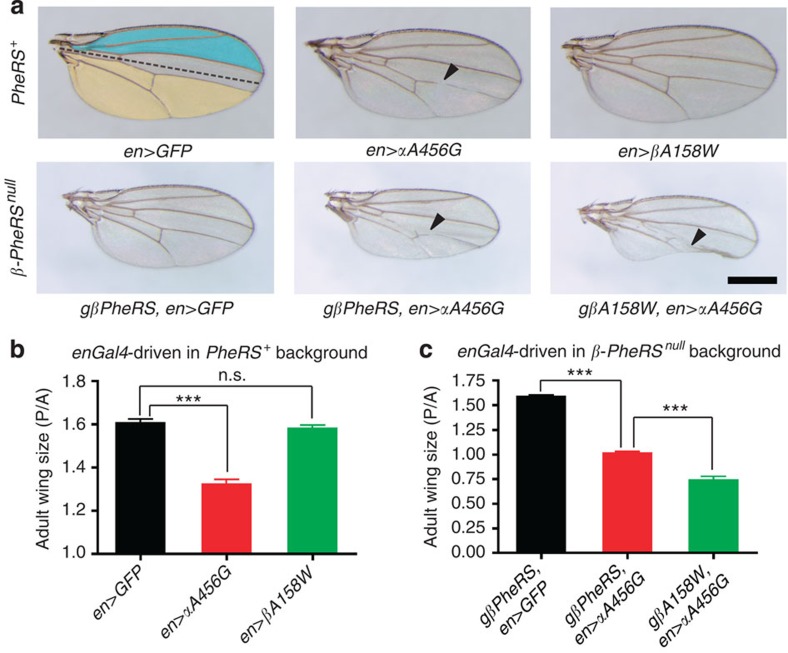
*PheRS-sd* mutants display reduced wing size. (**a**) Adult wing phenotypes induced by *PheRS-sd* mutations. *en-Gal4* (*en*) was used to drive transgene expression in the posterior compartment of developing wing discs. The dark dashed line indicates the border of the anterior/posterior compartment. In the *PheRS* wild-type background, wings expressing *GFP* and *βA158W* were normal. The ones expressing *αA456G* showed vein defects (arrowhead) and also reduced wing size in the posterior compartment. In the *gβPheRS*-rescued *β*-*PheRS*^*null*^ background, wings expressing *GFP* and αA456G, respectively, showed similar phenotypes as in the *PheRS*^+^ background. In contrast, wings expressing *αA456G* in a *gβA158W* background displayed severe size reduction with significant parts of the organ missing altogether. Scale bars represent 500 μm. (**b**,**c**) Quantification of wing sizes from the experiment shown in **a**. Twelve single female wings from different flies were analysed for each genotype. Wing size was measured by counting the pixels in an anterior (A; outlined in blue) and a posterior area (P; outlined in yellow) of each wing, and the ratio (P/A) was calculated. (**b**) In a *PheRS*^*+*^ wild-type background, *αA456G* mutants showed a size decrease, but *βA158W* did not. (**c**) In the rescued *β*-*PheRS* background, the *αA456G* mutant showed a size decrease and, when *αA456G* and *βA158W* are expressed, wing size was further decreased. Error bars represent s.e.m. *n*=12, n.s., not significant; ***P<0.001, *t*-test.

Translation is a major force for cellular growth. We therefore also assessed the effect of the *PheRS-sd* mutations on growth. The *Drosophila* wing is a proven system to study growth control *in vivo*. We used the *engrailed-Gal4* (*en-Gal4*) driver to express our test genes in the posterior compartment of the wing disc. This allowed us to compare the effect on the posterior cells to the wild-type control cells in the anterior compartment of the very same organ. As seen in Fig. 5a, expressing the *αA456G* mutation caused vein defects, while expression of the *βA158W* mutation did not cause any detectable wing phenotypes. However, expressing both, the mutant *αA456G* and the mutant *βA158W*, simultaneously in the posterior compartment caused a dramatic decrease in wing size, and in some wings entire regions of the posterior compartment were missing. This feature is reminiscent of the enhanced effect caused by expression of both mutations during eye development.

To quantify the changes in wing size, we compared the size of a clearly defined posterior region with such a region in the anterior compartment and calculated the ratio between them. The wild-type posterior/anterior (P/A) ratio was ~1.6. Expressing the *αA456G* mutation in the posterior compartment in the wild-type background reduced the wing size and the P/A ratio dropped to 1.3 (Fig. 5b). In rescued *β-PheRS* mutants expressing *αA456G* posteriorly, the ratio was ~1 (Fig. 5c). Wing size and ratio were further reduced (ratio <0.75) when *αA456G* was expressed in the posterior compartment in the *βA158W* background. These results demonstrate that the *PheRS-sd* mutations, and in particular the *αA456G* mutant, interfere with normal organ growth.

### *PheRS-sd* mutations induce apoptosis and reduce proliferation

**Figure 6:**
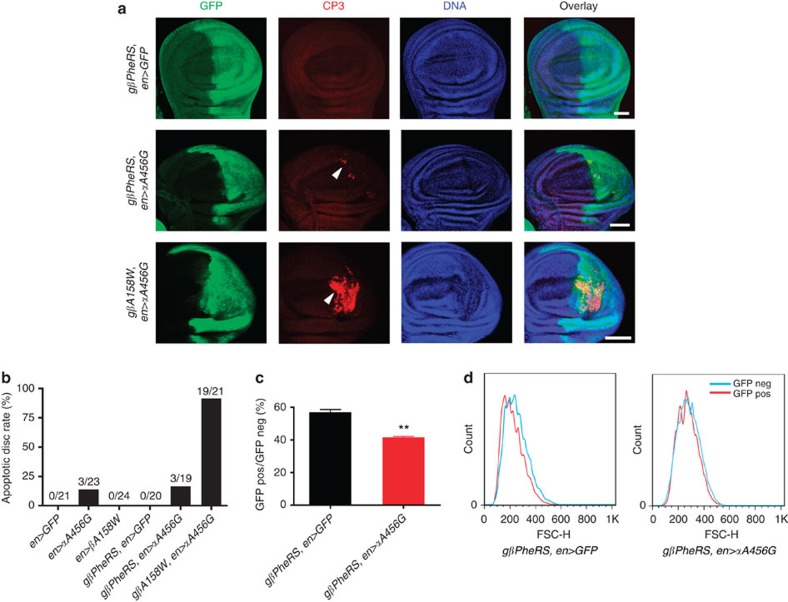
*PheRS-sd* mutations induce apoptosis and reduce cell proliferation. (**a**) Confocal microscopy images of wing discs from third instar wandering larvae. The posterior compartment is marked with GFP in green. CP3 is visualized in red with anti-CP3 antibodies. The anterior wing disc compartment (GFP-negative) serves as internal control. All flies shown are in the *β-PheRS*^*null*^ background, rescued by *gβPheRS* or *gβA158W*. A few CP3-positive cells were observed in *αA456G* mutant wing discs; however, many more cells underwent apoptosis when *αA456G* and *βA158W* mutants were expressed. Some CP3-positive cells are pointed out by arrowheads. Note that CP3-positive cells (red) always overlap with GFP (green). DNA is shown in blue. Scale bars represent 50 μm. (**b**) Quantification of apoptotic wing discs. No apoptosis was detectable in wing discs from controls and wing discs expressing only *βA158W*. A small number of wing discs expressing *αA456G* showed apoptotic cells. Apoptosis was most prominent in wing discs that expressed αA456G and βA158W mutant protein. (**c**,**d**) Wing discs were subjected to FACS analysis. The GFP signal was used to distinguish cell populations that express the transgenes. (**c**) The *αA456G* mutation reduces cell numbers. Cell numbers were determined relative to the cell numbers in the anterior (GFP-negative) area. Error bars indicate s.e.m. *n*=3, **P<0.01, *t*-test. (**d**) The *αA456G* mutation does not reduce cell size. Cell size was determined by Forward Scatter. Cells from the GFP-positive compartment are smaller than the ones from the GFP-negative compartment in the control. In *αA456G* mutants, their sizes are similar. Figure shows representatives of three independent experiments.

We next tested whether *PheRS-sd* mutations cause reduced wing size by inducing cell death. Staining wing discs for the CP3 epitope, we identified a small number of CP3-positive cells in cases where we expressed the *αA456G* mutation (Fig. 6a). Expressing the *αA456G* and the *βA158W* mutant simultaneously, we observed a much higher percentage of CP3-positive cells and some of these cells formed clusters rather than being dispersed over the disc. Importantly, these apoptotic cells were restricted to the posterior compartment where the *PheRS-sd* mutations were expressed. Confirming theses results with the TdT-mediated dUTP nick end labelling (TUNEL) assay (Supplementary Fig. 1), we conclude that apoptosis contributed to the impaired organ size.

To further evaluate the contribution of cell death to the phenotype, we quantified the number of wing discs that showed apoptosis in the staining assay (Fig. 6b). Wing discs from controls and wing discs expressing the *βA158W* mutant did not show signs of apoptosis. In contrast, wing discs expressing the *αA456G* and *βA158W* mutations simultaneously showed apoptosis in 19 out of 21 cases. Surprisingly, wing discs expressing the *αA456G* mutation alone showed very low levels of apoptosis (less than 16%), despite showing a significantly reduced wing size (compared with Fig. 5b,c). These data indicate that apoptosis mainly contributed to the size decrease in the wing when both mutant subunits were co-expressed. The smaller wing size observed in the *αA456G* appears to be more because of reduced cell proliferation, decreased cell size or both. To investigate this possibility, the same *en-Gal4* was used to drive *PheRS* gene expression and green fluorescent protein (GFP) was used to mark these cells. Cells from third instar larval wing discs were dissociated and analysed by FACS. The effect on cell proliferation was determined by calculating the ratio of number of cells from the posterior compartment (GFP-positive) to number of cells from the anterior compartment (GFP-negative). The FACS result showed that the *αA456G* mutant decreased this ratio from ~56% to 40% (Fig. 6c). Surprisingly, the cell size of the *αA456G* mutant was similar or slightly bigger than the wild-type cell size when compared by Forward Scatter (Fig. 6d). This analysis suggests that the additional contribution to the wing size reduction is reduced cell proliferation rather than decreased cell size. In conclusion, *PheRS-sd* mutations impair organ size by acting on cell proliferation and apoptosis. Apparently, the defect in the first sieve mainly affects cell proliferation, while defects in both sieves together further induce apoptosis.

### Sieving-defective mutants induce ER stress

**Figure 7:**
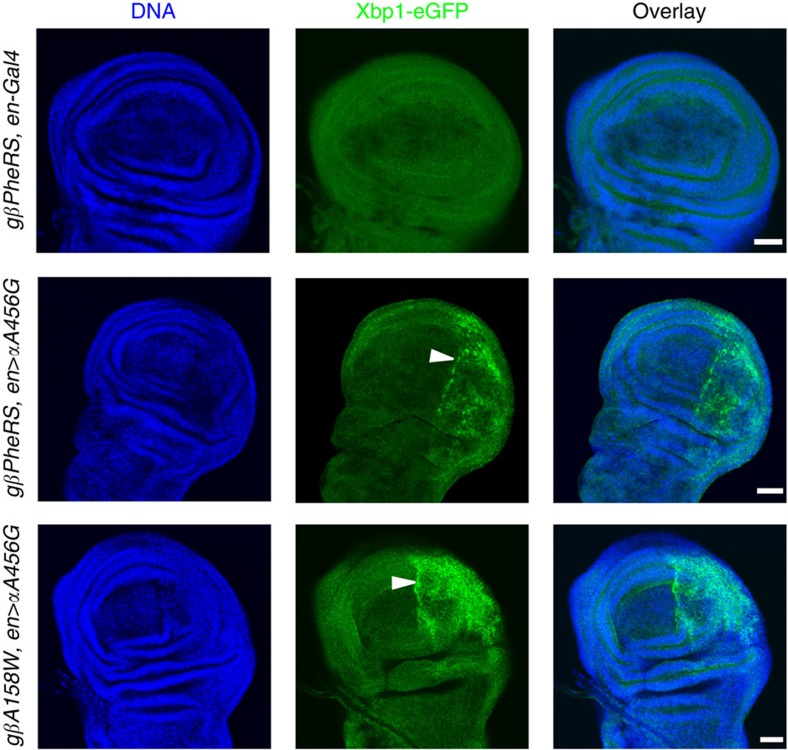
Sieving-defective mutations induce ER stress. An Xbp1-eGFP reporter was used to monitor the expression of the spliced Xbp1 isoform in *PheRS-sd* mutant wing discs using immunostaining. While no signal was observed in controls, GFP expression was detected both in *αA456G* mutants and in cells expressing the mutant forms *αA456G* and *βA158W*. Note that the GFP signal is always restricted to the posterior compartment. DNA is in blue and GFP is in green. Scale bars represent 25 μm.

The *PheRS αA456G* mutation is predicted to increase non-cognate amino-acid activation, and the *βA158W* mutant is not able to edit the faulty activation. As a consequence, non-cognate amino acids could be incorporated during protein synthesis, leading to the accumulation of misfolded proteins. In the lumen of the ER, misfolded proteins can impair the protein-folding capacity of the ER, a condition known as ER stress^35^,^36^,^37^. ER transmembrane receptors detect the onset of ER stress and initiate the unfolded protein response including cytoplasmic *Xbp1* mRNA splicing. To test whether the *PheRS-sd* mutations induce ER stress, we monitored *Xbp1* splicing with the Xbp1-eGFP (enhanced GFP) reporter^38^, in which the splicing-induced frameshift leads to eGFP expression. Wing discs expressing the *αA456G* mutant form or the *αA456G* and *βA158W* mutant forms of PheRS in the posterior compartment showed clear GFP expression above control levels in this compartment (Fig. 7). We also stained for the ER stress marker phosphorylated eukaryotic initiation factor 2α. Immunofluorescence analysis demonstrated that eukaryotic initiation factor 2α is transiently phosphorylated in several wing discs expressing both mutant forms together, but not in the ones expressing only the mutant *α-PheRS αA456G* in the posterior compartment or only the mutant *β-PheRS* in the anterior compartment (Supplementary Fig. 2). These results together revealed that the defect in the first sieve causes a certain level of ER stress and defects in both sieves induce strong ER stress.

### *PheRS-sd* mutants are sensitive to non-cognate amino acids

**Figure 8:**
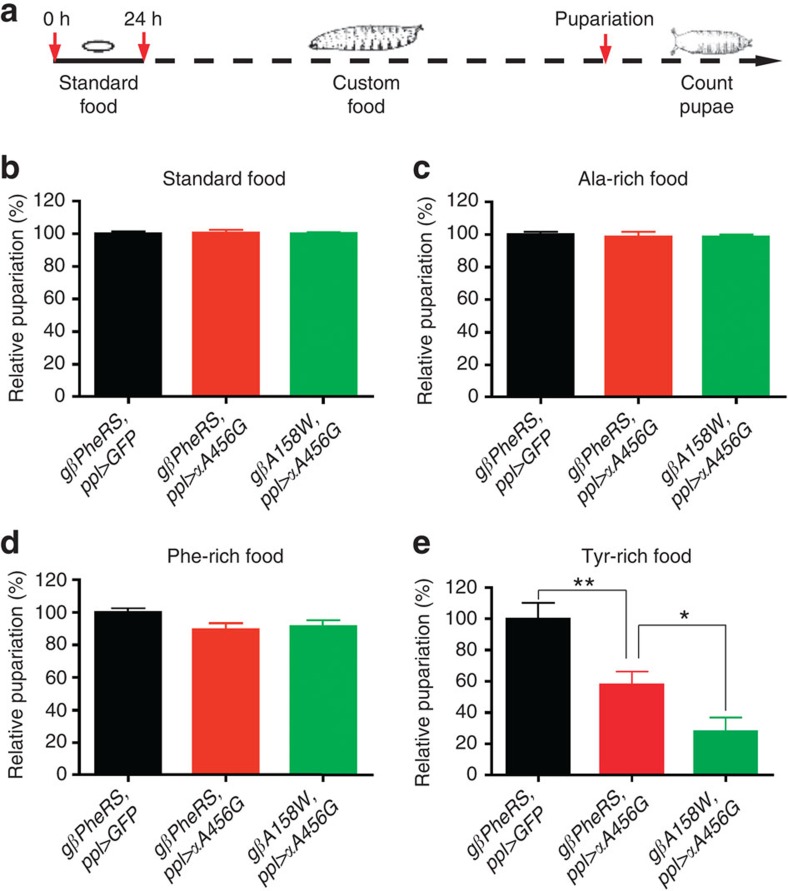
*PheRS-sd* mutant flies are sensitive to non-cognate amino acids. (**a**) Scheme illustrating the experimental procedure. The *ppl-Gal4* driver was used to drive *PheRS-sd* mutations in the larval fat body. Embryos were aged to 24 h after egg laying. First instar larvae with the correct genotype were selected and transferred to different amino-acid-rich food. When the larvae developed into pupae, the number of pupating individuals was recorded to calculate the pupariation rate. (**b**–**e**) *PheRS-sd* mutant flies are sensitive to the non-cognate amino acid Tyr. Larvae were raised on standard food (**b**), Ala-rich food (100 mM, **c**), Phe-rich food (100 mM, **d**) and Tyr-rich food (100 mM, **e**). No significant differences in pupariation rate were observed between control and *PheRS-sd* mutants on standard food and Ala- or Phe-rich food. On Tyr-rich food, the pupariation rate of the *αA456G* mutant was reduced to ~60%, and the one of larvae expressing *αA456G* and *βA158W PheRS* to ~30%. Error bars represent s.e.m. *n*=6. **P<0.01, *P<0.05, *t*-test.

Because of its similar structure Tyr, can be misactivated by PheRS. Expecting that *PheRS-sd* mutations increase the production of misacylated Tyr-tRNA^Phe^, we tested whether these mutations are sensitive to Tyr. Using *ppl-Gal4* we expressed the *PheRS-sd* mutations in the fat body, a tissue that has a high demand for nutrients and grows dramatically at the larval stage^39^. Larvae were challenged by the addition of different amino acids to the fly food and their pupariation rate was recorded (Fig. 8a). Reared on normal fly food, there was no difference in the pupariation rate between the wild type, *αA456G*-expressing larvae and larvae expressing mutant *αA456G* plus *βA158W PheRS* (Fig. 8b). In addition, no significant differences were observed between the Ala-rich and Phe-rich food (Fig. 8c,d). In contrast, Tyr-rich food reduced the pupariation rate of *αA456G*-expressing larvae to ~60%, and the rate of larvae expressing both mutant forms to ~30% of the wild type (Fig. 8e). This specific sensitivity suggests that these *PheRS* mutations are sieving-defective *in vivo*.

### *PheRS-sd* mutations promote F-to-Y substitution in proteins

**Figure 9:**
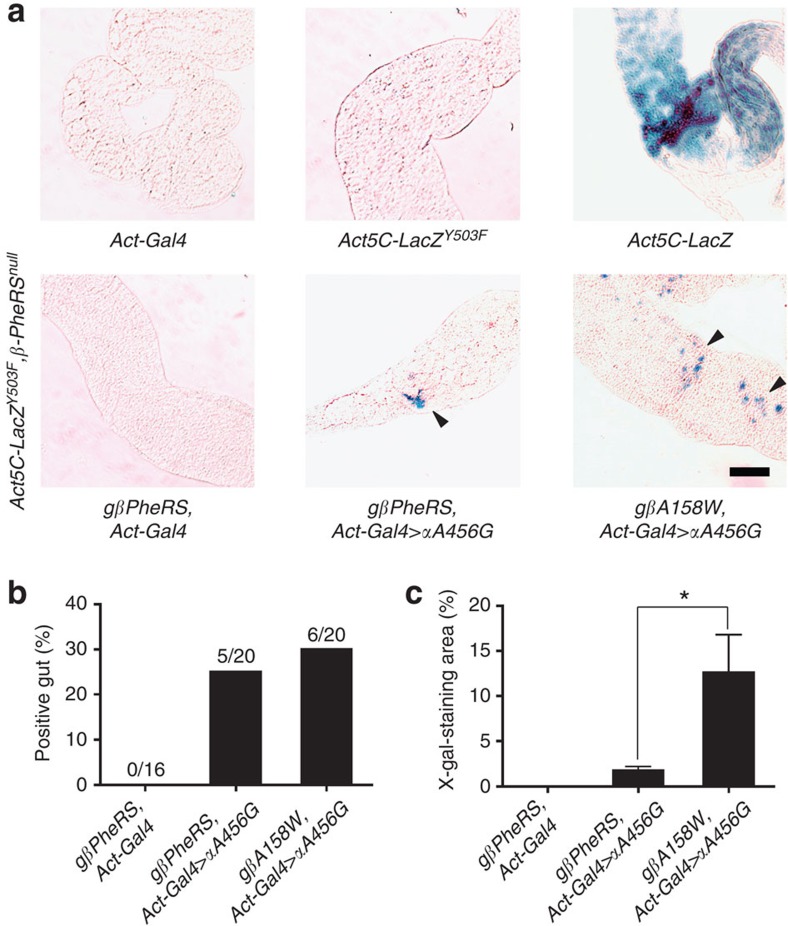
*PheRS-sd* mutations induce Phe-to-Tyr substitutions. (**a**) The FtoY reporter was generated to analyse the Phe-to-Tyr substitution *in vivo*. Third instar larvae were dissected and stained with X-gal. In driver-only larvae and in *LacZ*^*Y503F*^ mutants, no clear staining was detected, while a strong signal was observed in *LacZ* wild-type larval guts. *PheRS-sd* mutations restore β-galactosidase activity of *LacZ*^*Y503F*^ mutants. In the *β*-*PheRS*-rescued *β*-*PheRS*^*null*^ background, the *αA456G* mutant produced low levels of blue signal in the gut, and when *αA456G* and *βA158W PheRS* were expressed, stronger staining of additional cells become apparent (arrowheads). Scale bar represents 100 μm. (**b**) Quantification of number of guts that stained positive for X-gal. (**c**) Quantification of the blue staining region of the gut. The same size area of the gut was chosen for each sample and the blue pixels within this area were determined to calculate the ratio. Error bars indicate s.e.m. *n*=4, *P<0.05, *t*-test.

To measure misincorporation caused by *PheRS-sd* mutations *in vivo*, we generated a F-to-Y (FtoY) reporter using the bacterial LacZ protein. Tyr503 of the LacZ protein is crucial for its β-galactosidase activity. When this codon is mutated to a Phe codon, β-galactosidase activity decreases to less than 1% (refs 40, 41). We therefore expressed a mutant version of *LacZ*, *LacZ*^*Y503F*^, under the *Act5C* promoter in flies. Restored β-galactosidase activity in the fly tissue then indicates misincorporation of Tyr in position 503. As shown in Fig. 9a, in *PheRS* wild-type flies neither the *Act5C-LacZ*^*Y503F*^ nor the *Act-Gal4* driver control produced the blue staining indicative of β-galactosidase activity in the gut, while the wild-type *Act5C-LacZ* produced a strong staining. In *PheRS-sd* mutations the results were different. In the *αA456G* mutant, we detected some blue signal, but the staining was more intense in tissue expressing mutant *αA456G* plus *βA158W* PheRS. Although the frequency of staining guts remained similar for *αA456G*-expressing guts and guts expressing *αA456G* and *βA158W* mutant forms, interestingly, the latter showed a significant increase in the staining area (Fig. 9b,c). In summary, the assay of restoring the β-galactosidase activity in *LacZ*^*Y503F*^ demonstrates that *PheRS-sd* mutations lead to an enhanced Phe-to-Tyr substitution in proteins *in vivo*. With these results we shed light on the effects of sieving-defective PheRS on protein mistranslation, cellular stress and proper functioning of the cell.

## Discussion

Here we reported the generation and characterization of the first double-sieve animal model in *Drosophila*. The accuracy of PheRS in aminoacylation depends on the proper functioning of its double sieve, the amino-acid recognition sieve and the editing sieve^18^. Until now, animal models for studying the amino-acid double-sieving function of aaRSs in multicellular eukaryotes were rare. The high conservation of fundamental biological pathways and the powerful genetic tools make *Drosophila* a great system for such studies. We therefore generated the first double-sieving model in *D. melanogaster*. PheRS was our enzyme of choice because its amino-acid recognition and editing structure was well studied in single cell organisms^12^,^24^,^25^,^26^. Our analysis of the PheRS double-sieving model revealed many new aspects concerning functions of the amino-acid recognition sieve and the amino-acid editing sieve *in vivo*.

The editing function can be studied alone when the second sieve is defective while the amino-acid recognition sieve is intact. Surprisingly, the only phenotype we observed in the editing-defective fly mutants was a weak one, older adults showed advanced ageing (Fig. 4d). For several reasons, this effect does not appear to be caused by a reduced aminoacylation activity. The editing domain is physically separated from the activation site and the enzymatic aminoacylation activity measured *in vitro* was just as high as in the wild type (Fig. 2e). Therefore, the phenotypes of the editing mutant are most likely due to the editing defect. Accumulation of misfolded proteins results in chronic exposure to ER stress and probably affects the function of proteins with a slow turnover more severely. This could explain the advanced ageing observed in older flies. The observation that younger flies seem to be able to handle the editing defects better than older ones could also have to do with the animal’s ability to respond to ER stress. Recent research showed that in *Caenorhabditis elegans* this ability is lost with age^42^. While the editing defect in *Drosophila* PheRS causes only a relatively mild phenotype, a mutation in the AlaRS of mice, which results in a mild editing defect for Ser-tRNA^Ala^, induces more defects^43^. This mutant is the only sieving-defective animal model and it covers only the second sieve. AlaRS editing-defective mice exhibited smaller body size, patchy hair loss and, once they became older than 30 weeks, also a reduced latency time in the rotorod test, a sign of neurodegeneration. Interestingly, in the AlaRS mouse model, cellular phenotypes were only found in cerebellar Purkinje neuronal cells, but not in other proliferating cells. Clearly, different cell types, age groups and species can tolerate different levels of protein mistranslation^44^.

For the first time in a higher eukaryotic system our study also addressed the importance of the first sieve, the amino-acid recognition sieve. Strikingly, the change in this first sieve caused many defects, including loss of neuronal cells, impaired locomotive performance and smaller organ size even if the editing sieve was functional (Figs 3, 4, 5). Analysis of these phenotypes at the cellular level suggested that these defects were mainly caused by reduced cell proliferation and only low levels of apoptosis were detected (Fig. 6). The *in vitro* Phe aminoacylation assay showed reduced activity in the mutant with the defect in the first sieve (Fig. 2e), indicating that decreased aminoacylation and translation could be a reason for the effects observed at the cellular level. However, the *in vitro* results have to be interpreted with cautions. First, in contrast to *in vitro* assay, the enzymes in cells may not perform at their maximal activity. Second, heterozygous mutant flies are mostly normal, indicating that a 50% reduction of PheRS activity is tolerated without phenotypic consequences. Third, it was reported that most of PheRS (more than 90%) is bound to ribosomes in an RNA-dependent manner in human cells and only the remaining small fraction of free PheRS is thought to perform the aminoacylation function^45^. Furthermore, despite the high conservation of this residue during evolution, there is a natural Ala-to-Gly substitution in *Saccharomyces cerevisiae*, which still supports the high proliferation of the organism^44^. Therefore, it is likely that the *in vitro* Phe aminoacylation assay would overestimate the effect of the mutation on translation speed and phenotype.

The defect in the first recognition sieve clearly resulted in amino-acid misincorporation and induced ER stress in our fly model (Figs 7 and 9). This was surprising because in *in vitro* assays with the prokaryotic wild-type β-subunit, PheRS was fully capable of editing misacylated Tyr-tRNA^Phe^ (refs 12, 46). It thus seems that *in vivo* in flies the amount of misacylated Tyr-tRNA^Phe^ exceeded the editing capability of PheRS and therefore some Tyr became still incorporated into proteins (Fig. 9). Protein mistranslation initiates ER stress, and persistent ER stress can be the reason for reduced cell proliferation and apoptosis^35^. An interesting possibility is also that the defect in the first sieve may reduce the aminoacylation activity *in vivo* indirectly by engaging a large fraction of PheRS in editing activity and leaving too few PheRS enzymes to perform aminoacylation. In summary, the phenotypes caused by the defect in the amino-acid recognition sieve are the combined effects of reduced activity, overloading the correction mechanism and protein mistranslation.

Previous studies with defective double sieves were performed only in the single cell organism *E. coli*, where they showed growth retardation^24^. In flies, the double-sieving-defective mutants gave rise to strong and novel phenotypes. Ubiquitous expression caused lethality during development, while tissue-specific expression in eyes and wings led to smaller, defective organs, and to partial loss of these organs (Figs 3 and 5). Interestingly, our analysis of the double-sieving-defective mutant revealed a clear synergism between the two activities. At the cellular level, the ‘double mutant’ does not only reduce cell proliferation but also displays strong apoptosis, which can be induced by protein mistranslation and strong, persistent ER stress. It is also noteworthy that our experiments were mainly performed in proliferating cells, indicating that this double-sieving-defective model is not restricted to neuronal cells, but applies also to many types of cells and tissues.

Mutations in aaRS genes lead to many human diseases, mainly neuropathies and myopathies. For example, nine mutations in the *GlyRS* were reported to cause neurological diseases, including CMT disease type 2D and distal spinal muscular atrophy type V^6^. Mutations in TyrRS also cause CMT disease, which was successfully modelled in *Drosophila*^7^,^47^. However, little is known about the underlying mechanism. By generating and investigating the double-sieve model in higher eukaryotes, we addressed the importance of accurate aminoacylation in development and physiology. The fact that we discovered defects in several different tissues broadens the application of this model and widens the set of available tools.

For our study we changed one amino-acid residue for each sieve and these alterations to the binding pockets led to defective sieving functions. Several additional amino-acid residues make crucial contributions to the formation of the proper pocket and the sieve. For example, at least another six residues in the editing pocket of PheRS are important for the editing activity in archaea^25^. Similarly, we speculate that there are likely to be several residues involved in discriminating structurally similar amino acids in the first sieve. Interestingly, a recent study showed that not only gene mutations but also oxidative stress can inactivate a sieve^48^. Furthermore, the first sieve not only needs to be able to discriminate between the cognate and non-cognate L-amino acids but also between a variety of similar molecules such as metabolites, oxidative products and in the case of PheRS also the clinically used L-DOPA^49^. This and the fact that the first sieve is crucial for every aaRS makes this sieve a very attractive subject to study. Our double-sieving fly model greatly broadens our understanding of the double-step quality control of aminoacylation. It illustrates the importance of accurate aminoacylation and translational fidelity in development and physiology, and it also provides new explanations for human disease mechanisms.

## Methods

### PheRS DNA constructs and generation of transgenic flies

On the basis of the sequence information from FlyBase, the full-length cDNAs of *α-PheRS* (CG2263) and *β-PheRS* (CG5706) were obtained with RT–PCR and cloned into pUAST-attB^50^. Genomic sequences of both subunits, including their entire coding regions and also ~1 kb upstream and downstream sequences, were also amplified and cloned into pw^+^SNattB to generate the genomic rescue constructs^51^. Mutations of PheRS, as well as the C-terminal myc-tagged genomic *β-PheRS*, were created with the QuickChange Site-Directed Mutagenesis Kit (Stratagene). Constructs were verified by sequencing. The primer sequences used for DNA construction and double-stranded RNA (dsRNA) generation are listed (Supplementary Table 1). Transgenic flies were generated using the *φ*C31 integration system^52^.

### Fly stocks and genetics

Flies were kept on standard food and crosses were mostly performed at 25 °C unless otherwise noted. Fly strains used in this study were *α-PheRS*^*G2060*^ (BL no. 26625), *jar*^*322*^ (BL no. 8776), *Df(3R)Exel6198* (BL no. 7677), *engrailed-Gal4* (BL no. 30564), *eyeless-Gal4*(BL no. 5535) and *Act-Gal4* (BL no. 3954, BL no. 4414), *UAS-GFP* (BL no. 6658), *tub-Gal80*^*ts*^ (BL no. 7019), *UAS-RNAi-Cdk7* (VDRC no. 103413), *UAS-RNAi-α-PheRS* (VDRC no. 33514) and *UAS-RNAi-β-PheRS* (VDRC no. 42046). The *P[C95w+]* line^53^,^54^ was obtained from K.G. Miller, *ppl-Gal4* (ref. 39) from P. Léopold and the ER-stress marker *UAS-xbp1-eGFP*^38^ from H.D. Ryoo. To characterize the *PheRS* mutants, DNA from *α-PheRS*^*G2060*^ and *jar*^*322*^ was amplified and sequenced. *jar*^*322*^*/Df(3R)Exel6198* was used to generate *β-PheRS*^*null*^. For long-term experiments with these flies, the *jar* gene *P[C95w+]* was introduced into this background. The genotypes of flies used in each figure are listed in Supplementary Table 2.

### *Drosophila* β-PheRS antibody generation

A C-terminal 14 amino-acid peptide (CSRDDIGRKLNKNI) was synthesized and used to immunize rabbits to generate anti-β-PheRS polyclonal antibodies (GenScript). The antisera were affinity-purified against full-length β-PheRS fused to the maltose-binding protein at its N terminus. On western blots, the antibodies recognized a β-PheRS band around 66 kDa.

### Protein preparation and aminoacylation assay

*Drosophila* PheRS proteins were expressed and purified^55^. Wild-type and mutant *α-PheRS* cDNAs were subcloned into the pET-28a plasmid (Novagen), and wild-type and mutant *β-PheRS* cDNAs were subcloned into the pET LIC (2A-T) plasmid (Addgene). The N-terminal His-tagged α-PheRS and untagged β-PheRS were co-expressed in the *E. coli* strain Rosetta (Novagen) with 1 mM isopropylthiogalactoside induction at 25 °C for 15 h. Proteins were purified with Ni-NTA affinity resin (Qiagen), and the concentration was determined using bovine serum albumin (BSA) as a standard. The aminoacylation assay was performed at 25 °C in a 100-μl reaction mixture containing 50 mM Tris-HCl (pH 7.5), 10 mM MgCl_2_, 4 mM ATP, 5 mM 2-mercaptoethanol, 100 μg ml^−1^ BSA, 3 U ml^−1^ *E. coli* carrier tRNA, 5 μM [^3^H]-amino acid (L-Phe or L-Tyr) and 1 μM tRNA^Phe^ from brewer’s yeast (Sigma). The reaction was initiated by adding 0.8 μM PheRS. In each experiment, a 15-μl aliquot was removed at six different incubation time points, spotted on filter discs and washed three times with ice-cold 5% trichloroacetic acid and once with ice-cold ethanol. After filter discs were dried, the radioactivity was measured by scintillation counting. An enzyme-free reaction was used to measure the background, which was then subtracted from the measured values.

### *Drosophila* cell culture and RNAi treatment

*Drosophila* Kc cells were incubated at 25 °C in Schneider’s *Drosophila* medium supplemented with 10% heat-inactivated fetal calf serum (FCS) and 50 μg ml^−1^ penicillin/streptomycin. Standard protocols were followed for the general maintenance. To induce RNAi knockdown in *Drosophila* cells, dsRNA treatment was performed^56^. dsRNA ~500 bp in length was generated with the RNAMaxx High Yield Transcription Kit (Stratagene). Cells were diluted to a concentration of 10^6^ cells ml^−1^ in serum-free medium, and dsRNA was added directly to the medium at a concentration of 15 μg ml^−1^. Cells were incubated for 1 h, followed by addition of FCS-containing medium. Subsequently, cells were kept in the incubator to allow for turnover of the target protein. Unless indicated otherwise, cells were then harvested 3 days after dsRNA treatment.

### Western blots and *in situ* staining experiments

For western blots, extracted proteins were resolved with SDS–PAGE and transferred to polyvinylidene difluoride membranes. Blots were blocked, probed with primary antibodies overnight at 4 °C and then probed with secondary antibodies for 1 h at room temperature^57^. Detection was performed with the LI-COR Odyssey system. Uncropped blots for Fig. 1 are provided as Supplementary Fig. 3. For immunostaining, tissues were dissected in PBS and collected on ice for a maximum of 30 min. After 30-min fixation with 4% paraformaldehyde, samples were blocked with 5% non-fat milk for 2 h at room temperature. Incubation with primary antibodies was performed at 4 °C overnight, followed by secondary antibody incubation for at least 2 h at room temperature^58^. For TUNEL staining, fixed samples were incubated with Terminal Transferase (Roche Diagnostics) and DIG-11-dUTP (Roche Diagnostics) for 1.5 h at room temperature. Hoechst 33258 (2.5 μg ml^−1^) was added in a washing step and the samples were mounted in Aqua-Poly/Mount (Polysciences). Stained samples were imaged with a Leica TCS-SP2 confocal laser-scanning microscope and processed with the Photoshop software. Antibodies used for western blots and immunostaining are listed in Supplementary Table 3.

### Retina semithin sections

Adult *Drosophila* retina semithin sections were prepared as follows^59^. Fly heads were dissected and fixed with 2% glutaraldehyde and post-fixed with 2% OsO_4_. After dehydration through an ethanol series, the samples were embedded in Durcupan resin (Sigma). Fly heads were trimmed with a Leica microtome to obtain 0.5- to 1-μm-thick retina sections. The sections were stained with 1% Toluidine-blue and analysed with light microscopy.

### Wing disc dissociation and FACS analysis

Wing disc dissociation^60^ was performed with wandering third instar larvae derived from 4-h egg collections. Larvae were dissected in 1 × Hank’s buffered salt solution (HBSS) for a maximum of 30 min. Around 20 wing discs were incubated with gentle agitation for ~2 h in 500 μl 10 × Trypsin-EDTA supplemented with 50 μl 10 × HBSS and 10 μl Vybrant DyeCycle Ruby stain (Molecular Probes). Dissociated cells from wing discs were directly analysed with FACS-Calibur flow cytometer (Becton Dickinson). Data were analysed with the FlowJo software.

### Climbing assays and lifespan analysis

For climbing assays^61^,^62^, 20 adult male flies were briefly anaesthetized with CO_2_ and placed in a 100-ml glass measuring cylinder fitted with a cotton plug on top. The cylinder was divided into five regions with region 5 being at the top and 1 at the bottom. After a 1-h recovery, cylinders were knocked on a soft surface to force flies to the bottom. Flies were then given 20 s to climb towards the top of the column. At this point they obtained points according to the region they reached. Each assay was repeated three times with 5-min intervals. Results were averaged and the average points gave the locomotive index. For each genotype, a minimum of 60 flies was tested. Lifespan^63^ was determined at 25 °C with a 12-h light/dark cycle. Flies were reared at standard larval density. Flies emerging over a 3-day period were collected and subjected to experiment. Female and male flies were separated and maintained in vials at a density of 10 flies per vial. Flies were transferred to fresh vials every 2–3 days and scored for deaths. One hundred fifty flies were used per genotype. The Prism GraphPad software was used for data analysis.

### Amino-acid sensitivity assay

Amino-acid-rich food was prepared by addition of 100 mM amino acids (L-Ala, L-Phe and L-Tyr) to the same volume of *Instant Drosophila Medium* (Formula 4-24, Carolina). Eggs were laid in a 2-h period on standard *Drosophila* food and allowed to develop for 24 h. Thirty to fifty larvae of the desired genotype were then transferred to one vial containing the amino-acid-rich food. Number of pupae was recorded for each vial.

### FtoY reporter and X-gal staining

The LacZY503F mutation was used to generate the FtoY reporter. *LacZ* was amplified from the pMT/V5-His/LacZ plasmid (Invitrogen), and the *LacZ*^*Y503F*^ mutation was created with the QuickChange Site-Directed Mutagenesis Kit (Stratagene). The *Act5C* promoter, amplified from Ac5-STABLE2-neo plasmid (Addgene), was fused to wild-type *LacZ* and to *LacZ*^*Y503F*^ to drive their expression. The two fragments were then cloned into pw^+^SNattB vector, and transgenic flies were generated. To assay β-galactosidase activity, X-gal staining was performed. Late third instar larvae were dissected and fixed in 4% paraformaldehyde for 10 min. After washing, the samples were incubated in X-gal staining solution (1 × PBS, 1 mM MgCl_2_, 5 mM K_4_Fe(CN)_6_, 5 mM K_3_Fe(CN)_6_, 0.3% Triton X-100) with 1.0 mg ml^−1^ X-gal and 1.0 mg ml^−1^ nitroblue tetrazolium at 37 °C for the appropriate time. Samples were then washed, dissected and mounted for microscopic analysis.

## Author contributions

J.L. and B.S. conceived the ideas and designed the experiments. J.L. conducted most experiments and performed the analysis of the results. M.B. and A.W. contributed to characterize PheRS fly mutants and developed the β-PheRS antibody. J.L. and B.S. wrote the manuscript.

## Additional information

**How to cite this article:** Lu, J. *et al*. Double-sieving-defective aminoacyl-tRNA synthetase causes protein mistranslation and affects cellular physiology and development. *Nat. Commun.* 5:5650 doi: 10.1038/ncomms6650 (2014).

## Supplementary Material

Supplementary Information Supplementary Figures 1-3, Supplementary Tables 1-3

Supplementary Movie 1 Negative geotaxis climbing assays are performed to examine fly locomotive performance. Flies expressing *αA456G* mutant (right cylinder) displayed decreasing climbing ability compared with the ones expressing the wild type *α-PheRS* (left cylinder).
