# Supplementary material for: Double-sieving-defective aminoacyl-tRNA synthetase causes protein mistranslation and affects cellular physiology and development
Source: Nat Commun. 2014 Nov 27;5:5650. doi: 10.1038/ncomms6650 (PMC4263187; doi:10.1038/ncomms6650)
Supplement: Supplementary Information — Supplementary Figures 1-3, Supplementary Tables 1-3 [file ncomms6650-s1.pdf]

Supplementary Fig. 1

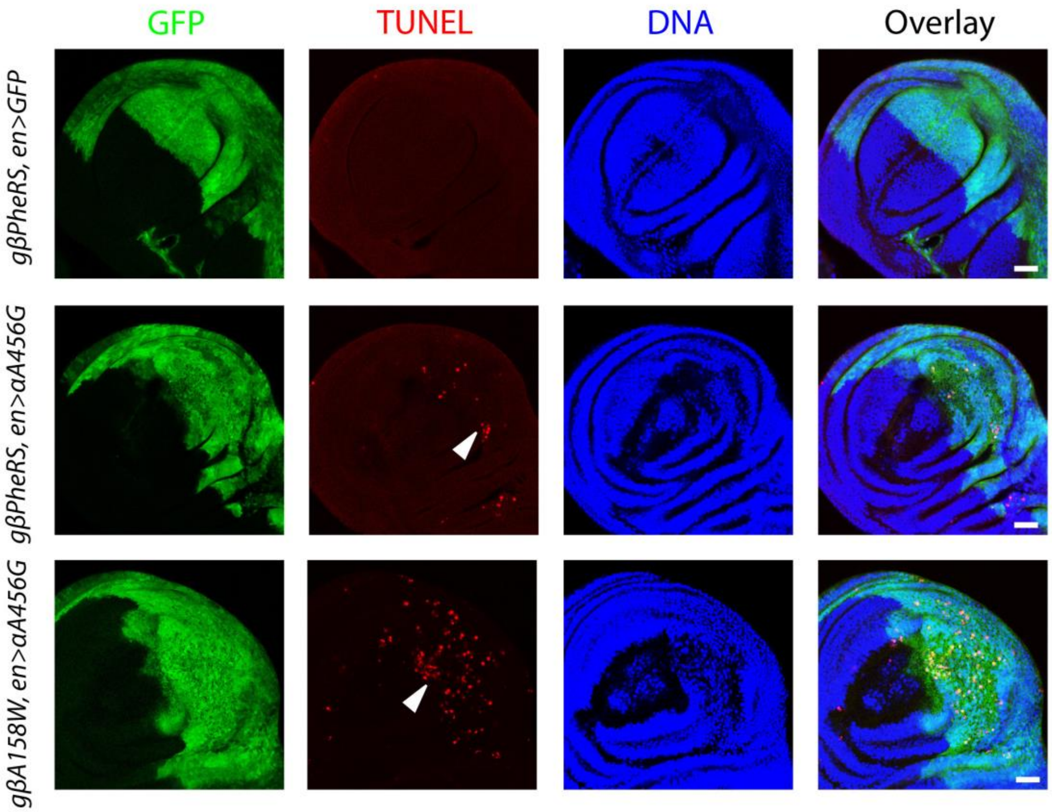

Supplementary Fig. 1. TUNEL staining of wing discs from 3rd instar wandering larvae. GFP in green marks the posterior compartment, and the anterior wing disc compartment (GFP-negative) serves as internal control. TUNEL is shown in red. A few TUNEL-positive cells underwent apoptosis in *αA456G* mutant wing discs, and many more cells were observed when *αA456G* and *βA158W* mutants were expressed. Some TUNEL-positive cells are pointed out by arrowheads. Note that few developmental apoptotic cells were also detected. DNA is in blue. Scale bars represent 25 μm.

Supplementary Fig. 2

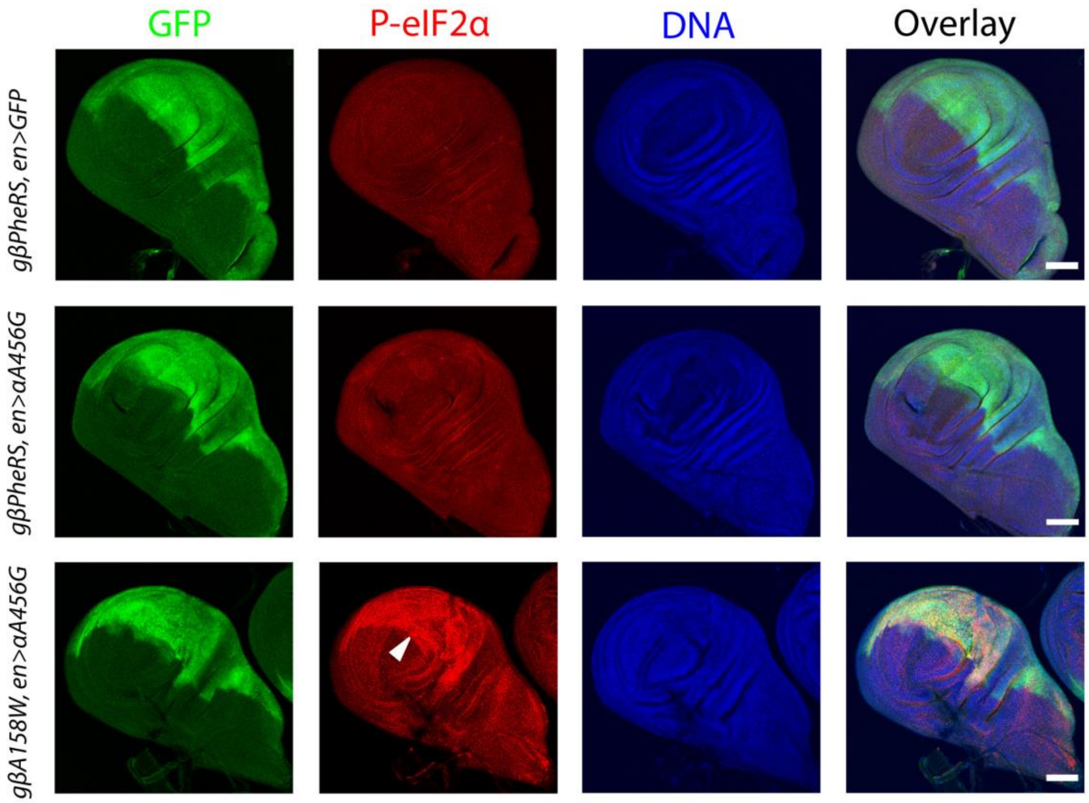

Supplementary Fig. 2. Immunostaining of phosphorylated eukaryotic initiation factor 2α (eIF2α) in late 3rd instar larval wing discs. No clear signal was detected in controls and *αA456G* single mutants, while phosphorylation of eIF2α (red) was observed in mutants expressing *αA456G* and *βA158W*. GFP (green) marks the posterior compartments where the UAS-transgenes are expressed, and the phospho-eIF2α signal always overlapped with the elevated GFP signal. DNA is in blue. Scale bars represent 50 μm.

Supplementary Fig. 3

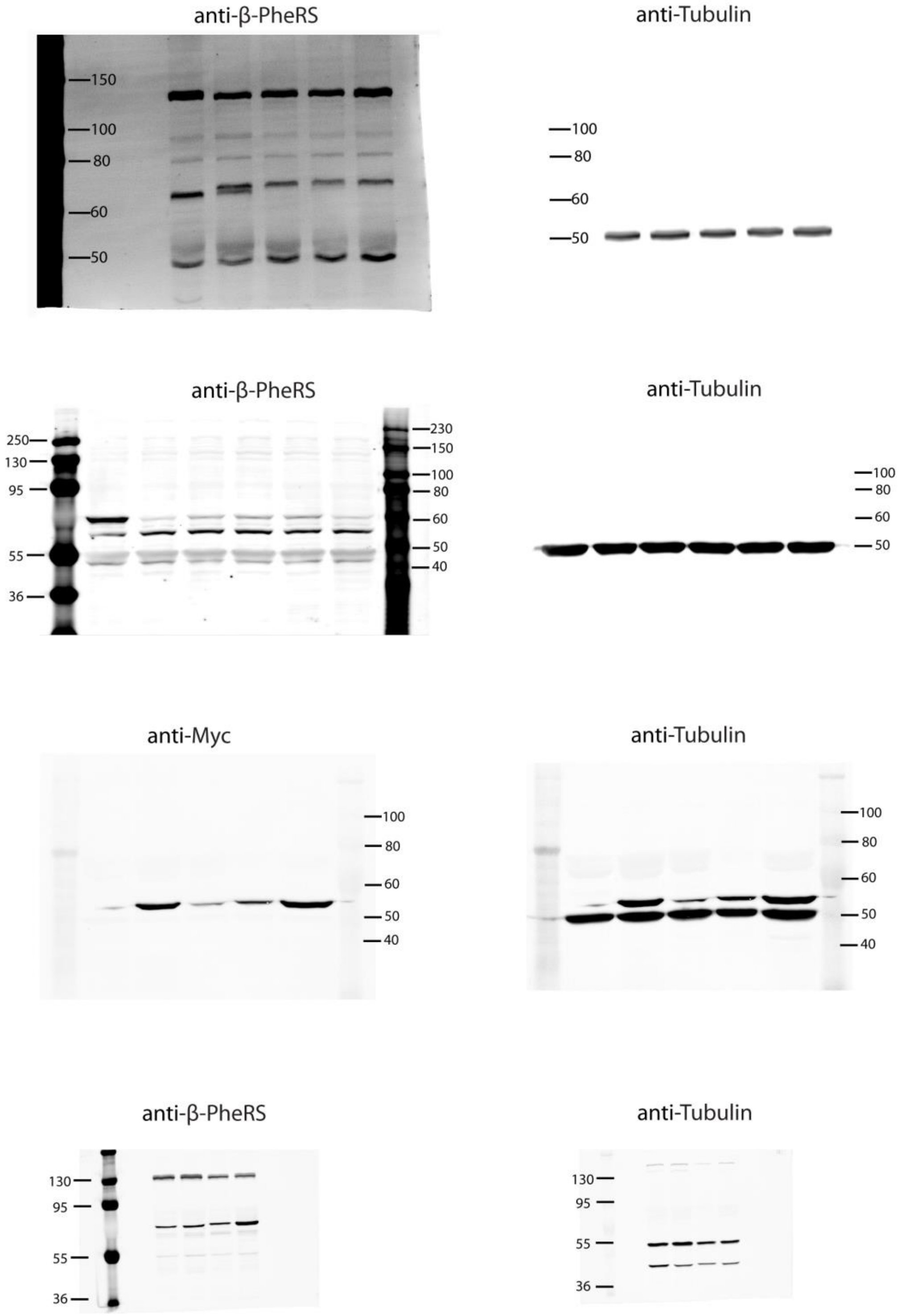

Supplementary Fig. 3. Uncropped Western blots shown in Fig. 1.

Supplementary Table 1

Supplementary Table 1: The sequences of primer used in this study.

Name	Sequence (5' to 3')	Application
gβPheRS-fwd	AGCGGAACGTGAAACTAACGAAG	Genomic rescue
gβPheRS-rev	AGCTTTCGAACTGGAAGGCAAAGAACCGCAAG	construct of *β-PheRS*
gαPheRS-fwd	CGCGGATCCATCCGGCGAGAGAGTGTCTTTG	Genomic rescue
gαPheRS-rev	CGGGGTACCTATGCCTGGCGATAATCGTG	construct of *α-PheRS*
βPheRS-fwd	TCACGAGGTACCATGCCCACCATTGGAGTTAAACG	Overexpression
βPheRS-rev	TGACCGCTCGAGTTAGACGAAAGGCTCAATGGTG	construct of *β-PheRS*
αPheRS-fwd	ATAAGATAGCGGCCGCATGCATCCCGATCTCACG	Overexpression
αPheRS-rev	TGCTAGTCTAGATTAAGCATGATCCAGCCGGCA	construct of *α-PheRS*
gβPheRS-myc-fwd	CCATTGAGCCTTTCGTCGGATCCGGATCTGAGCAAAAGCT	Genomic construct of
	CATTTCTGAAGAGGACTTGTAAAAGATTTGCAAAATG	c-term myc tagged *β-*
gβPheRS-myc-rev	CATTTTGCAAATCTTTTACAAGTCCTCTTCAGAAATGAGCTTTTGCTC	*PheRS*
	AGATCCGGATCCGACGAAAGGCTCATTGG	
myc-αPheRS-fwd	ATAAGATAGCGGCCGCATGGAGCAAAAGCTCATTTCTGA	Overexpression
	AGAGGACTTGGGATCCGGATCTCATCCCGATCTCACGGAG	construct of n-term
myc-αPheRS-rev	TGCTAGTCTAGATTAAGCATGATCCAGCCGGCA	myc tagged *α-PheRS*
αA456G-fwd	CGTGAATGTAATCGGCTGGGGTCTGTCGC	*α-PheRS A456G*
αA456G-rev	GCGACAGACCCCAGCCGATTACATTCACG	mutation construct
βA158W-fwd	CGGAAGCGTACTCTGGTGTGGATCGGAACGCACGATCTG	*β-PheRS A158W*
βA158W-rev	CAGATCGTGCGTTCCGATCCACACCAGAGTACGCTTCCG	mutation construct
Ri-Amp^R-fwd	TAATACGACTCACTATAGGGAGGCCAGAAACGCTGGTGAAAGT	*Amp*^{R} (mock) dsRNA
Ri-Amp^R-rew	TAATACGACTCACTATAGGGAGGGATACGGGAGGGCTTACCAT	synthesis
Ri-βPheRS-fwd	TAATACGACTCACTATAGGGAGGGCCAATCATTCGGGAATCA	*β-PheRS* dsRNA
Ri-βPheRS-rev	TAATACGACTCACTATAGGGAGGAGGCAGGGACTTCTTAATGT	synthesis
Ri-αPheRS1-fwd	TAATACGACTCACTATAGGGAGGCAAGAAACGCAAGCTCCTC	*α-PheRS*^{1} dsRNA
Ri-αPheRS1-rev	TAATACGACTCACTATAGGGAGGGAACTCCGCCAGATGTGTG	synthesis
Ri-αPheRS2-fwd	TAATACGACTCACTATAGGGAGGATTTGGAGACCACCGACAAG	*α-PheRS*^{2} dsRNA
Ri-αPheRS2-rev	TAATACGACTCACTATAGGGAGGAATCCATTTGCGAGCATTTC	synthesis
Ri-αPheRS3-fwd	TAATACGACTCACTATAGGGAGGTCAGCATCGACAAGGTGTTC	*α-PheRS*^{3} dsRNA
Ri-αPheRS3-rev	TAATACGACTCACTATAGGGAGGTTAGATCCACTTTGGGTCCG	synthesis
PE-αPheRS-fwd	GGGAATTCCATATGCATCCCGATCTCACG	Protein expression
PE-αPheRS-rev	ATAAGATAGCGGCCGCTTAAGCATGATCCAGCCGGCA	construct of *α-PheRS*
PE-βPheRS-fwd	TATGGCGCGCCATGCCCACCATTGGAGTTAAACG	Protein expression
PE-βPheRS-rev	TCACGAGGTACCTTAGACGAAAGGCTCAATGGTG	construct of *β-PheRS*
LacZ-Y503F-fwd	GATATTATTTGCCCGATGTTCGCGCGCGTGGATGAAGAC	Construct of LacZ
LacZ-Y503F-rev	GTCTTCATCCACGCGCGCGAACATCGGGCAAATAATATC	Y503F mutation
LacZ-fwd	CCGGGGTACCATGATAGATCCCGTCGTTTTACAAC	LacZ/Y503F DNA
LacZ-rev	GCGCGGATCCTTATTTTTGACACCAGACCAACTG	amplification
ActLacZ-fwd	AAGCCCAAGCTTTAAAAAAAATCATGAATGGCATC	Act-LacZ/Y503F
ActLacZ-rev	AAGGATAGAAGCGGCCGCACTGATCATAATCAGCCATACCAC	DNA amplification

Supplementary Table 2

Supplementary Table 2: Fly genotypes in each figure.

Figure	Genotype
Fig. 1c<br>(left to right)	(1), *w*^{1118} as wild type (*wt*)<br>(2), *w*; *β-PheRS::myc*; *Df(3R)Exel6198/TM3,Sb*<br>(3), *w*; *β-PheRS::myc/+*; *β-PheRS*^{1}*/Df(3R)Exel6198*
Fig. 1e<br>(left to right)	(1), *w*; *ppl-Gal4/ UAS-RNAi-Cdk7*; *UAS-myc::αPheRS/+*<br>(2), *w; ppl-Gal4/ UAS-RNAi-αPheRS*; *UAS-myc::αPheRS/+*<br>(3), *w; ppl-Gal4/+*; *UAS-myc::αPheRS/UAS-RNAi-βPheRS*
Fig. 1f<br>(left to right)	(1), *w*; *ppl-Gal4/+*; *UAS-GFP/+*<br>(2), *w; ppl-Gal4/+*; *UAS-αPheRS/+*<br>(3), *w; ppl-Gal4/UAS-βPheRS*; *+*<br>(4), *w; ppl-Gal4/UAS-βPheRS*; *UAS-αPheRS/+*
Fig. 3a, 3c<br>(top left to<br>bottom right)	(1), *w*; *ey-Gal4/+*; *UAS-GFP/+*<br>(2), *w*; *ey-Gal4/+*; *UAS-αA456G/+*<br>(3), *w*; *ey-Gal4/UAS-βA158W*; *+*<br>(4), *w*; *ey-Gal4/gβPheRS*; *UAS-GFP, β-PheRS*^{1}*/Df(3R)Exel6198*<br>(5), *w*; *ey-Gal4/gβPheRS*; *UAS-αA456G, β-PheRS*^{1}*/Df(3R)Exel6198*<br>(6), *w*; *ey-Gal4/gβA158W*; *UAS-αA456G, β-PheRS*^{1}*/Df(3R)Exel6198*
Fig. 4a, 4c	Control: *w*; +; *Act-Gal4/UAS-αPheRS*<br>Mutant *αA456G*: *w*; +; *Act-Gal4/UAS-αA456G*
Fig. 4b, 4d	Control: *w*; *gβPheRS/P[C95w*$^{+}$*]*; *β-PheRS*^{1}*/Df(3R)Exel6198.*<br>Mutant *βA158W*: *w*; *gβA158W/P[C95w*$^{+}$*]*; *β-PheRS*^{1}*/Df(3R)Exel6198*
Fig. 4e<br>(top to bottom)	(1), *w*; *tub-Gal80*^{ts}*/gβPheRS*; *β-PheRS*^{1}*/Act-Gal4, Df(3R)Exel6198*<br>(2), *w*; *tub-Gal80*^{ts}*/gβPheRS*; *UAS-αA456G, β-PheRS*^{1}*/Act-Gal4, Df(3R)Exel6198*<br>(3), *w*; *tub-Gal80*^{ts}*/gβA158W*; *UAS-αA456G, β-PheRS*^{1}*/Act-Gal4, Df(3R)Exel6198*
Fig. 5a<br>(top left to<br>bottom right)	(1), *w*; *en-Gal4/+*; *UAS-GFP/+*<br>(2), *w*; *en-Gal4/+*; *UAS-αA456G/+*<br>(3), *w*; *en-Gal4/UAS-βA158W*; *+*<br>(4), *w*; *en-Gal4/gβPheRS*; *UAS-GFP, β-PheRS*^{1}*/Df(3R)Exel6198.*<br>(5), *w*; *en-Gal4/gβPheRS*; *UAS-αA456G, β-PheRS*^{1}*/Df(3R)Exel6198*<br>(6), *w*; *en-Gal4/gβA158W*; *UAS-αA456G, β-PheRS*^{1}*/Df(3R)Exel6198*
Fig. 6b<br>(left to right)	(1), *w*; *en-Gal4/+*; *UAS-GFP/+*<br>(2), *w*; *en-Gal4/+*; *UAS-αA456G/UAS-GFP*<br>(3), *w*; *en-Gal4/UAS-βA158W*; *UAS-GFP/+*<br>(4), *w*; *en-Gal4/gβPheRS*; *β-PheRS*^{1}*/UAS-GFP, Df(3R)Exel6198.*<br>(5), *w*; *en-Gal4/gβPheRS*; *UAS-αA456G, β-PheRS*^{1}*/UAS-GFP, Df(3R)Exel6198*<br>(6), *w*; *en-Gal4/gβA158W*; *UAS-αA456G, β-PheRS*^{1}*/UAS-GFP, Df(3R)Exel6198*

Fig. 6c-d	(1), *w*; *en-Gal4/gβPheRS*; *β-PheRS*^{1}*/UAS-GFP, Df(3R)Exel6198*<br>(2), *w*; *en-Gal4/gβPheRS*; *UAS-αA456G, β-PheRS*^{1}*/UAS-GFP, Df(3R)Exel6198*
Fig. 7<br>(top to bottom)	(1), *w*; *en-Gal4, UAS-Xbp1-eGFP/gβPheRS*; *β-PheRS*^{1} */Df(3R)Exel6198*<br>(2), *w*; *en-Gal4, UAS-Xbp1-eGFP/gβPheRS*; *UAS-αA456G, β-PheRS*^{1} */Df(3R)Exel6198*<br>(3), *w*; *en-Gal4, UAS-Xbp1-eGFP/gβA158W*; *UAS-αA456G, β-PheRS*^{1} */Df(3R)Exel6198*
Fig. 8	(Black), *w*; *ppl-Gal4/gβPheRS*; *UAS-GFP, β-PheRS*^{1}*/Df(3R)Exel6198*<br>(Red), *w*; *ppl-Gal4/gβPheRS*; *UAS-αA456G, β-PheRS*^{1} */Df(3R)Exel6198*<br>(Green), *w*; *ppl-Gal4/gβA158W*; *UAS-αA456G, β-PheRS*^{1} */Df(3R)Exel6198*
Fig. 9a<br>(top left to bottom right)	(1), *w*; *Act-Gal4/+*<br>(2), *w*; +; *Act5c-LacZ*^{Y503F}<br>(3), *w*; +; *Act5c-LacZ*<br>(4), *w*; *Act-Gal4/gβPheRS*; *UAS-GFP, β-PheRS*^{1}*/Act5c-LacZ*^{Y503F}*, Df(3R)Exel6198*<br>(5), *w*; *Act-Gal4/gβPheRS*; *UAS-αA456G, β-PheRS*^{1}*/Act5c-LacZ*^{Y503F}*, Df(3R)Exel6198*<br>(6), *w*; *Act-Gal4/gβA158W*; *UAS-αA456G, β-PheRS*^{1}*/Act5c-LacZ*^{Y503F}*, Df(3R)Exel6198*
Sup. Fig. 1<br>Sup. Fig. 2<br>(top to bottom)	(1), *w*; *en-Gal4/gβPheRS*; *β-PheRS*^{1}*/UAS-GFP, Df(3R)Exel6198.*<br>(2), *w*; *en-Gal4/gβPheRS*; *UAS-αA456G, β-PheRS*^{1}*/UAS-GFP, Df(3R)Exel6198*<br>(3), *w*; *en-Gal4/gβA158W*; *UAS-αA456G, β-PheRS*^{1}*/UAS-GFP, Df(3R)Exel6198*

Supplementary Table 3

Supplementary Table 3: Antibodies used in the study.

Antibodies	Product information	Dilution
anti-β-PheRS	Self made	1:1,000
anti-Tubulin	Developmental Studies Hybridoma Bank, AA4.3	1:2,000
anti-c-myc	Developmental Studies Hybridoma Bank,9E10	1:5
IRDye 680RD anti-rabbit	LI-COR, #926-68071	1:15,000
IRDye 800CW anti-mouse	LI-COR, #926-32210	1:15,000
anti-Cleaved Caspase-3	Cell Signaling, #9661	1:200
anti-phospho-eIF2α	Cell Signaling, #3597	1:150
anti-Elav	Developmental Studies Hybridoma Bank, 9F8A9	1:50
anti-GFP	ImmunoKontact, #042704	1:300
anti-DIG	Roche, #11333089001	1:1,000
Alexa Fluor 594 anti-rabbit	Molecular Probes, A-11012	1:1,000
Alexa Fluor 488 anti-rabbit	Molecular Probes, A-11008	1:1,000
Alexa Fluor 488 anti-mouse	Molecular Probes, A-11001	1:1,000
Cy3 anti-sheep	Jackson ImmunoResearch, #713-165-147	1:500
